# Primary aldosteronism is associated with risk of urinary bladder stones in a nationwide cohort study

**DOI:** 10.1038/s41598-021-86749-3

**Published:** 2021-04-08

**Authors:** Mu-Chi Chung, Cheng-Li Lin, Ming-Ju Wu, Cheng-Hsu Chen, Jeng-Jer Shieh, Chi-Jung Chung, Chi-Yuan Li, Tung-Min Yu

**Affiliations:** 1 Division of Nephrology, Department of Medicine, Taichung Veterans General Hospital, 1650 Taiwan Boulevard Sect. 4, Taichung, 40705 Taiwan, ROC; 2 Ph.D. Program in Translational Medicine, National Chung Hsing University, Taichung, Taiwan; 3 Rong Hsing Research Center for Translational Medicine, National Chung Hsing University, Taichung, Taiwan; 4 Management Office for Health Data, China Medical University and Hospital, Taichung, Taiwan; 5 Institute of Biomedical Sciences, National Chung Hsing University, Taichung, Taiwan; 6 Department of Education and Research, Taichung Veterans General Hospital, Taichung, Taiwan; 7 Department of Public Health, College of Public Health, China Medical University, Taichung, Taiwan; 8 Department of Medical Research, China Medical University Hospital, Taichung, Taiwan; 9 Graduate Institute of Biomedical Sciences and School of Medicine, College of Medicine, China Medical University, Taichung, Taiwan

**Keywords:** Endocrinology, Nephrology, Urology

## Abstract

We analyzed database from the Taiwan National Health Insurance to investigate whether primary aldosteronism (PA) increases the risk of bladder stones. This retrospective nationwide population-based cohort study during the period of 1998–2011 compared patients with and without PA extracted by propensity score matching. Cox proportional hazard models and competing death risk model were used to estimate the hazard ratios (HRs), sub-hazard ratios (SHRs) and corresponding 95% confidence intervals (CIs). There were 3442 patients with PA and 3442 patients without PA. The incidence rate of bladder stones was 5.36 and 3.76 per 1000 person-years for both groups, respectively. In adjusted Cox hazard proportional regression models, the HR of bladder stones was 1.68 (95% CI 1.20–2.34) for patients with PA compared to individuals without PA. Considering the competing risk of death, the SHR of bladder stones still indicates a higher risk for PA than a comparison cohort (SHR, 1.79; 95% CI 1.30–2.44). PA, age, sex, and fracture number were the variables significantly contributing to the formation of bladder stones. In conclusion, PA is significantly associated with risk of bladder stones.

## Introduction

Primary aldosteronism (PA), with aldosterone hypersecretion, was found in 5–13% of patients with resistant hypertension^1^. In addition to its detrimental effect on the cardiovascular system, it is suggested that PA could adversely affect the process of mineral bone homeostasis. For example, an increased risk of osteoporosis and bone fractures was demonstrated in PA by the following mechanism: enhanced calcium excretion through renal tubules in urinary and intestinal cells. Meanwhile, a remarkably higher secondary secretion of parathyroid hormone is occasionally observed concomitantly with PA^2^. Additionally, other systemic effects that result from PA were reported previously, such as increased incidence of new-onset diabetes mellitus and metabolic syndrome^3,4^.

There is a 7–13% lifetime risk of developing nephrolithiasis; this results in not only significant morbidity but also substantial economic costs^5^. The stones in urolithiasis are comprised of calcium oxalate or phosphate compounds. In addition to traditional risk factors such as age, sex, race, geography, and high body mass index, other factors, including hyperparathyroidism, osteoporosis, and diabetes mellitus are highly likely to be associated with the development of nephrolithiasis^6^.

A previous study suggests that recurrent calcium nephrolithiasis was likely associated with PA through increased hypercalciuria and hypocitraturia and that the status of hypersecretion of urinary calcium can be attenuated after adrenalectomy^7,8^. In addition, excretion of acidic urine resulting from higher aldosterone levels might also promote formation of urate stones^9^.

Although bladder stones are uncommon in nephrolithiasis (estimated at 5%), such calculi are reported to result in multiple major complications such as acute urinary retention, dysuria, and even acute renal failure^10^. Bladder stones are not only the cause of several upper urinary tract stone diseases, but also urinary stasis caused by bladder outlet obstruction or benign prostate hyperplasia^11,12^. Some evidence suggests that aldosterone plays a key role in urinary bladder function. Aldosterone could affect calcium-activated potassium (BK) channels, which are essential in regulating the function of urinary bladder smooth muscle^13^ by potassium depletion. Therefore, it is reasonable to suppose that PA is likely to be associated with urinary stone risk.

To the present, data regarding the risk of developing bladder stones in PA are still lacking. The present study is devoted to investigating the association of bladder stones and PA in a national cohort with a long observational period.

## Results

Table 1 shows the PA and comparison cohort characteristics. Median age was 49.4 and 52.6 years for the PA and comparison cohorts, respectively (SMD, 0.04). In the PA and comparison cohorts, 46.5% and 58.3% of the patients were men, respectively (SMD, 0.24). The median frequency of medical visits was 0.54 and 0.62 for the PA and comparison cohorts, respectively (SMD, 0.08). Except for fracture, there was no difference in comorbidity percentage between the PA and comparison cohort.

**Table 1:** Comparison of demographic characteristics and comorbidity between PA versus a non-PA cohort by propensity score matching method.

	Primary aldosteronism				Standardized differences
	No		Yes		
	(n = 3442)		(n = 3442)		
	n	%	n	%	
Age, median (IQR)	52.6	34.5	49.4	21.7	0.04
**Stratify age**					
≤ 49	1580	45.9	1773	51.5	0.11
50–65	811	23.6	973	28.3	0.11
> 65	1051	30.5	696	20.2	0.24
**Gender**					0.24
Women	1434	41.7	1840	53.5	
Men	2008	58.3	1602	46.5	
Frequency of medical, visits/per year, median (IQR)	0.62	1.19	0.54	0.79	0.08
**Comorbidity**					
Hypertension	1392	40.4	1388	40.3	0.002
Diabetes	461	13.4	406	11.8	0.05
Hyperlipidemia	293	8.51	268	7.79	0.03
Gout	151	4.39	116	3.37	0.05
Urinary tract infection	349	10.1	272	7.90	0.08
Obesity	16	0.46	16	0.46	0.00
Chronic kidney disease	43	1.25	45	1.31	0.01
Fracture	709	20.6	266	7.73	0.38
Hematuria	38	1.10	17	0.49	0.07

*IQR* interquartile range, *PA* primary aldosteronism.

**Figure 1:**
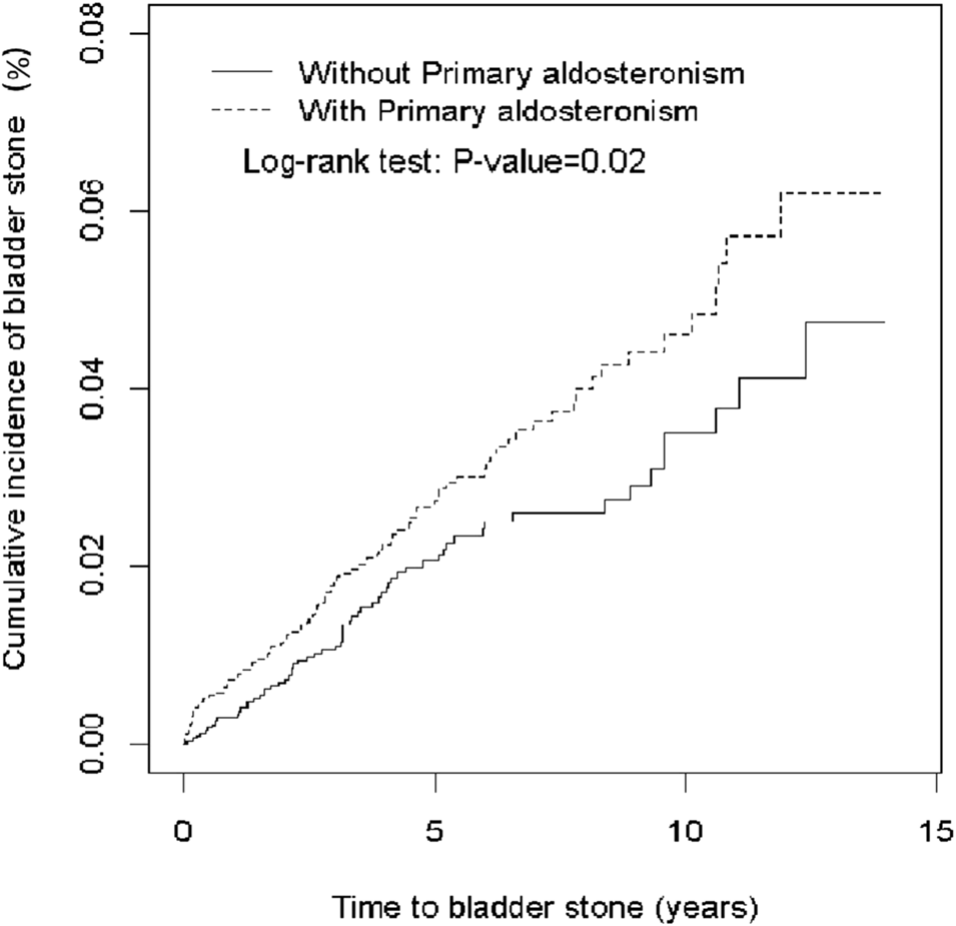
Cumulative incidence rate (%) of bladder stones in the PA cohort and the comparison cohort during the period of 1998-2011.

Table 2 demonstrated the incidence, HRs, and SHRs of bladder stones between the PA and comparison cohort. The overall bladder stone incidences were 5.36 and 3.76 per 1000 person-years for the PA and comparison cohorts, respectively. Figure 1 shows the incidence rate of bladder stones was greater for the PA cohort than the comparison cohort (log-rank test, P = 0.02). After adjusting for age, sex, and comorbidities, the HR of bladder stones was 1.68 (95% CI 1.20–2.34) for patients with PA compared to individuals without PA. Relative to individuals without PA, the HRs for bladder stones was 1.86 (95% CI 1.11–3.10), 1.18 (95% CI 0.64–2.16), and 2.07 (95% CI 1.05–4.07) for patients with PA who were 20–49 years of age, 50–64 years of age, and ≥ 65 years of age, respectively. Only in men, patients with PA were significantly associated with a higher risk of bladder stone development than those of the comparison cohort (HR 1.65; 95% CI 1.09–2.49).

**Table 2:** Comparison of bladder stone risk in incidence densities, hazard ratio, and sub-hazard ratio in a competing risk (death) model between patients with and without PA stratified by demographic characteristics and comorbidity.

	Primary aldosteronism						Crude HR^a^ (95% CI)	Adjusted HR^b^ (95% CI)	p-value	Crude SHR^†^ (95% CI)	Adjusted SHR^&^ (95% CI)	p-value
	No (n = 3442)			Yes (n = 3442)								
	Event	PY	Rate^#^	Event	PY	Rate^#^						
All	64	17,012	3.76	90	16,786	5.36	1.43 (1.03,1 .96)	1.68 (1.20, 2.34)	0.002	1.56 (1.15, 2.13)	1.79 (1.30, 2.44)	< 0.001
**Age**												
20–49	27	9261	2.92	45	9872	4.56	1.57 (0.97, 2.53)	1.86 (1.11, 3.10)	0.02	1.38 (0.85, 2.24)	1.53 (0.93, 2.54)	0.10
50–64	21	3688	5.69	25	4416	5.66	1.00 (0.56, 1.78)	1.18 (0.64, 2.16)	0.59	1.04 (0.58, 1.86)	1.23 (0.68, 2.24)	0.50
65 +	16	4063	3.94	20	2498	8.00	2.03 (1.05, 3.92)	2.07 (1.05, 4.07)	0.03	2.31 (1.18, 4.49)	2.38 (1.20, 4.72)	0.01
**Gender**												
Women	19	7300	2.60	37	9234	4.01	1.54 (0.89, 2.68)	1.74 (0.98, 3.07)	0.06	1.66 (0.98, 2.79)	1.83 (1.07, 3.14)	0.03
Men	45	9712	4.63	53	7552	7.02	1.51 (1.02, 2.25)	1.65 (1.09, 2.49)	0.02	1.67 (1.14, 2.46)	1.65 (1.09, 2.49)	0.02
**Comorbidity**^**§**^												
No	18	7843	2.29	47	9517	4.94	2.11 (1.23, 3.64)	2.18 (1.26, 3.78)	0.006	2.04 (1.21, 3.44)	2.12 (1.27, 3.57)	0.004
Yes	46	9168	5.02	43	7269	5.92	1.18 (0.78, 1.79)	1.27 (0.83, 1.93)	0.27	1.37 (0.92, 2.06)	1.42 (0.92, 2.19)	0.11

Rate^#^, incidence rate, per 1000 person-years.

Crude HR^a^, relative hazard ratio.

Adjusted HR^b^, adjusted hazard ratio, was calculated by Cox model and adjusted for age, sex, frequency of medical visits, and comorbidities of hypertension, diabetes, hyperlipidemia, gout, urinary tract infection, obesity, chronic kidney disease, fracture, and hematuria.

Crude SHR^†^, relative sub-hazard ratio.

Adjusted SHR^&^, adjusted sub-hazard ratio, was calculated by competing risk (death) model and adjusted for age, sex, frequency of medical visits, and comorbidities of hypertension, diabetes, hyperlipidemia, gout, urinary tract infection, obesity, chronic kidney disease, fracture, and hematuria.

Comorbidity^§^: Patients with any one of the comorbidities (including hypertension, diabetes, hyperlipidemia, gout, urinary tract infection, obesity, chronic kidney disease, fracture, and hematuria) were classified as the group of comorbidity.

*CI* confidence interval, *HR* hazard ratio, PY, *SHR* sub-hazard ratio.

Table 2 also included the competing risk of death; the SHR of bladder stones still indicated a higher risk for the PA and comparison cohorts (SHR, 1.79; 95% CI 1.30–2.44). In study subjects aged ≥ 65 years, the PA cohort was associated with a 2.38-fold higher risk of bladder stone development than the comparison cohort (SHR, 2.38; 95% CI 1.20–4.72). Compared to patients without PA, the SHRs of bladder stones were 1.83 (95% CI 1.07–3.14) and 1.65 (95% CI 1.09–2.49) for patients with PA in women and in men, respectively. The risk of bladder stones was significantly higher for PA patients than comparisons (SHR = 2.12, 95% CI 1.27–3.57) to individuals without any comorbidity.

Table 3 shows the results of univariable and multivariable competing risk between the PA and comparison cohorts. The significant results for single variable analysis were PA (SHR, 1.56; 95% CI 1.15–2.13), sex (women vs men SHR, 1.89; 95% CI 1.37–2.59), age (SHR, 1.02; 95% CI 1.02–1.03), and fracture (SHR, 1.65; 95% CI 1.09–2.48). The multivariable model, which involved PA, age, sex, and fracture, showed that all variables were significantly at higher risk of developing bladder stones.

**Table 3:** SHR and 95% CI of bladder stones in association with sex, age, and comorbidities in univariable and multivariable competing risk (death) model.

Variable	Crude		Adjusted^†^	
	SHR	(95% CI)	SHR	(95% CI)
Primary aldosteronism	1.56	(1.15, 2.13)**	1.79	(1.30, 2.44)***
Sex (women vs men)	1.89	(1.37, 2.59)***	1.88	(1.37, 2.59)***
Age, years	1.02	(1.02, 1.03)***	1.01	(1.01, 1.02)***
**Baseline comorbidities (yes vs no)**				
Hypertension	1.15	(0.81, 1.64)		
Diabetes	1.28	(0.80, 2.06)		
Hyperlipidemia	1.45	(0.86, 2.46)		
Gout	1.57	(0.80, 3.08)		
Urinary tract infection	1.37	(0.81, 2.33)		
Obesity	1.78	(0.25, 12.7)		
Chronic kidney disease	0.55	(0.08, 3.96)		
Fracture	1.65	(1.09, 2.48)	1.77	(1.16, 2.69)***
Hematuria	0.87	(0.12, 6.26)		

Crude SHR represents a relative sub-hazard ratio.

*CI* confidence interval, *SHR* sub-hazard ratio.

^†^The multivariable models included all statistically significant risk factors in the univariable competing risk (death) model.

*P < .05, **P < .01, ***P < .001.

A sensitivity analysis was performed with PA versus a non-PA cohort by 1:4 frequency matching based on age, sex, and all comorbidities (Supplementary Table 1). Supplementary Table 2 demonstrated the incidence, HRs, and SHRs of bladder stones between the PA and comparison cohort. After adjusting for age, sex, and comorbidities, the HR of bladder stones was 1.63 (95% CI 1.28–2.08) for patients with PA compared to individuals without PA. In this sensitivity analysis, outcome was also consistent with the primary analysis.

## Discussion

To the present, this is the first large-scale study to discover that PA is significantly associated with a higher bladder stones risk which was unrecognized previously. The findings disclosed an incidence rate of 5.36 per 1000 person-years of bladder stones in our PA cohort (cumulative incidence around 0.06% even after 10 years follow up) compared with that was 3.76 per 1000 person-years in control group and suggest that bladder stone was very easily ignored in daily practice because of a relative rare condition^14^. In our study, the bladder stone risk is high in patients with PA; particularly in those without any underlying disease which reached a 2.12-fold increased risk and that indicates a potential linkage between hyperaldosteronism and bladder stone formation.

In addition, in multivariable analysis of Cox regression model with adjusting associated confounders including gender, age, co-morbidities showed that fracture was significantly associated with a 1.77-fold increased risk of bladder stone which may seem a predictive factor for urinary stone. Of note, a lower proportion of cases with fracture were observed in patients with PA while compared to control group; nevertheless, our findings remained to exhibit a significantly higher risk of bladder stone in patients with PA. The findings implicate that PA may likely attribute to urinary stone directly independent of fracture. At last, in the sensitive analysis with frequency matching PA and non-PA group with sex, age and all comorbidities (including fracture), the findings were consistent. Taken together, the findings suggest that PA contributed to bladder stone formation by the disease per se rather than other factors.

Accumulating biologic evidence suggests that PA may play an important role in the development of nephrolithiasis. First, PA likely induces hypercalciuria through body volume expansion, which decreases both proximal tubule sodium and calcium absorption^15^. Second, PA induces hypocitraturia^7^. Potassium depletion in PA may induce proximal tubule intracellular acidosis and also cause hypocitraturia. These electrolyte disorders caused by PA itself could lead to the tubular abnormality which indirectly increases the formation of bladder stones. Moreover, PA might cause urinary bladder dysfunction because of hypokalemia. Hypokalemia is known as a cause of hypotonic bladder^16,17^. Several families of potassium channels, especially large-conductance voltage and calcium-activated BK channels, are essential to control urinary bladder smooth muscle contraction^13,18^.

PA was also suggested to be associated with new-onset diabetes mellitus and metabolic syndrome^3,4^. Both diabetes mellitus and metabolic syndrome induce more acidic urine to enhance urate crystallization and promote urate stone formation. Next, PA is accompanied by sympathetic overactivity^19^ and likely to aggravate urinary stasis^20^. At last, PA might be associated with benign prostatic hypertrophy (BPH), through elevated serum aldosterone^21^ and increased level of oxidative stress^22,23^.

Taken together, our findings provide clinical evidence that patients with chronically persistent hyperaldosteronism eventually develop bladder stones. This is supported by experimental data previously reported. Our findings showed that fracture history or female sex was also among the risk factors contributing to bladder stone formation. Previous study has suggested that bone fracture is significantly associated urinary tract stone^24,25^. Fracture, as a risk factor for bladder stones, suggests a role of metabolic abnormalities in the formation of bladder stones. Generally speaking, male sex is considered a traditional risk factor in the development of bladder stones, but this was not seen in our PA study cohort. This may be the result of the differences in the sexes that already exist in PA. For example, compared to male patients, female patients are more vulnerable to PA and associated with a higher risk of bilateral PA^26^, fractures^2^, depression mood, and even worse physical conditions^27^, all of which could partly explain why the female sex is a risk factor for the formation of bladder stones. The detailed mechanisms involved in the differences in bladder stone formation among the sexes in patients with PA need to be further clarified.

Some limitations to our study should be addressed. First, laboratory data could not be obtained from the database. Hence, metabolic factors associated with stone formation, such as serum glucose and electrolyte profiles, could not be further evaluated and may bias the findings. Proximity for these confounders, such as diabetes mellitus and hyperlipidemia, were adjusted as best we could in the study. Second, any occurrence of stone formation before the index date were excluded from our study; therefore, the case numbers could be underestimated. Third, influence of medication such as diuretics and potassium or calcium supplements could not be evaluated in our study. Forth, we could not distinguish patients with PA and unilateral hyperplasia from those with bilateral adenomas. Last, one limitation was inherent in the national inpatient database, which included only five diagnoses at discharge^28^, and some comorbidities might be under-estimated. Therefore, a relative lower incidence rates of hypertension either case group or control group was found after matching process.

In conclusion, the present study shows that patients with PA are at a significantly higher risk for the development of bladder stones which has never been reported previously. In addition to the adverse outcomes of cardiovascular disease and metabolic disorders in patients with PA, they have increased risk of bladder stones as well. Whether or not the suppression effect of mineralocorticoid receptor antagonists could decrease the risk of bladder stones should be further analyzed in the future.

## Materials and methods

The NHIRD encrypts patient personal information to protect privacy and provides researchers with anonymous identification numbers associated with relevant claims information. Patient consent is not required to access the NHIRD. This study was approved to fulfill the condition for exemption from the Institutional Review Board (IRB) of China Medical University (CMUH-104-REC2-115). The IRB of China Medical University (CMUH-104-REC2-115) waived the informed consent requirement. Our research was performed in accordance with relevant guidelines/regulations.

### Data sources

We used the National Health Insurance Research Database (NHIRD) to construct the study. The NHIRD contains the health insurance claim data from those insured through the Taiwan National Health Insurance program (Taiwan NHI). The Taiwan NHI is a national-based single payer health insurance program that has been in existence since 1995 and covered over 99% of 23 million Taiwan citizens in 1998. The health insurance claims data contains information including a registry of beneficiaries, disease record files (listed by the International Classification of Diseases, Ninth Revision, Clinical Modification [ICD-9-CM]), and other medical services. In this study, all disease records were collected from inpatient files. Before the government released the disease records, patient personal identification information was obscured for purposes of this research.

### Study population

We designed a retrospective, population-based, matched cohort study. We planned to build a cohort of patients with PA (ICD-9-CM 255.1) and a comparison cohort. The PA cohort involved patients with new-onset PA ˃18 years of age and initially diagnosed between the years 1998 and 2011. The index date for patients with PA was the day of first diagnosis. We excluded any patients with PA who also had a history of bladder stones (ICD-9-CM 592.0, 592.1, 592.9) before the index date. The candidate comparison sample included patients from the NHIRD who were not diagnosed with PA. The propensity score matching method was used in this study. We assigned equivalent index dates for candidate comparisons and PA cases. The propensity score of the PA and candidate comparisons was calculated using logistic regression models which involved the variables age, sex, frequency of medical visits, hypertension (ICD-9-CM 401-405), diabetes mellitus (ICD-9-CM 250), hyperlipidemia (ICD-9-CM 272), gout (ICD-9-CM 274), urinary tract infection (ICD-9-CM 599.0), obesity (ICD-9-CM 278.0), chronic kidney disease (CKD, ICD-9-CM 585), fracture (ICD-9-CM 800-829), and hematuria (ICD-9-CM 599.7). For each PA case, the matched comparison subjects were selected using the nearest propensity score by algorithm (http://www2.sas.com/proceedings/sugi29/165-29.pdf). The comparisons also excluded patients with a diagnosis of bladder stones before the index date. The main outcome of this study is that patients with PA have a higher risk for the development of bladder stones. Follow-up visits for these patients began on the index date and terminated when the patients either (1) lost their health insurance; (2) died; (3) were diagnosed with bladder stones; or (4) until December 31, 2013.

### Statistical analysis

Age distribution was expressed as mean ± SD; sex and comorbidities were expressed as number and percentage between the PA and comparison cohorts. The standardized mean difference (SMD), a test for distribution differences between the PA and comparison cohorts, was calculated from a difference in means or proportions of a variable divided by a pooled estimate of the SD of that variable. The significance level of SMD was set at a value over 0.1. The incidence density of bladder stones for the PA and comparison cohorts was calculated from the total number of bladder stone events divided by the sum of person time (per 1000 person-years). The cumulative bladder stone incidence curves for individuals with and without PA were drawn using the Kaplan–Meier method; the log-rank test was used to assess the difference in curves. To ascertain the risk of bladder stones between the PA and comparison cohorts, we used the single variable and multivariable Cox proportional hazard models to estimate the hazard ratios (HRs) and corresponding 95% confidence intervals (CIs). Because a death event could bias the estimation of bladder stone occurrence, we also used the competing risk model, a model developed from a standard Cox model^29^. We included the effect of competing death, shown as the sub-hazard ratios (SHRs) and 95% CIs of the bladder stone incidence in the PA and comparison cohorts. The data management and statistical analysis was performed using SAS 9.4 software (SAS Institute, Cary, NC, United States) and the incidence curve was created using R software^30^. The significance level was set at less than 0.05 for 2-sided testing.

## Supplementary Information

Supplementary Information
