# Supplementary material for: Primary aldosteronism is associated with risk of urinary bladder stones in a nationwide cohort study
Source: Sci Rep. 2021 Apr 8;11:7684. doi: 10.1038/s41598-021-86749-3 (PMC8032702; doi:10.1038/s41598-021-86749-3)
Supplement: Supplementary file 1 — Supplementary Information [file 41598_2021_86749_MOESM1_ESM.docx]

**Primary Aldosteronism is Associated with Risk of Urinary Bladder Stones in a Nationwide Cohort Study**

Mu-Chi Chung, MD ^1,2,3^; Cheng-Li Lin, MSc ^4^; Ming-Ju Wu, MD, PhD ^1^; Cheng-Hsu Chen, MD ^1^; Jeng-Jer Shieh, PhD ^3,5,6^; Chi-Jung Chung, PhD ^7,8^; Chi-Yuan Li, MD^9^; Tung-Min Yu, MD, PhD ^1,9^ ^*^

^1^Division of Nephrology, Department of Medicine, Taichung Veterans General Hospital, Taichung, Taiwan

^2^Ph.D. Program in Translational Medicine, National Chung Hsing University

^3^Rong Hsing Research Center For Translational Medicine, National Chung Hsing University

^4^Management Office for Health Data, China Medical University and Hospital, Taichung, Taiwan

^5^Institute of Biomedical Sciences, National Chung Hsing University, Taichung, Taiwan

^6^Department of Education and Research, Taichung Veterans General Hospital, Taichung, Taiwan

^7^Department of Health Risk Management, College of Public Health, China Medical University, Taichung, Taiwan

^8^Department of Medical Research, China Medical University Hospital, Taichung, Taiwan

^9^Graduate Institute of Biomedical Sciences and School of Medicine, College of Medicine, China Medical University, Taichung, Taiwan

Address correspondence to Tung-Min Yu M.D., PhD

Address: 1650 Taiwan Boulevard Sect. 4, Taichung, Taiwan 40705, ROC

E-mail: taichungnephrologist@gmail.com

Tel: +886-4-23592525 ext. 3048 FAX: 886-4-2359-5046

Supplement Table 1. Comparison of demographic characteristics and comorbidity between PA versus a non-PA cohort by 1:4 frequency matching based on age, sex, and comorbidities.

|  | Primary Aldosteronism | | | | *p*-value |
| --- | --- | --- | --- | --- | --- |
|  | No | | Yes | |  |
|  | (n=13760) | | (n=3442) | |  |
|  | n | % | n | % |  |
| Age, median (IQR) | 49.4  (40.0-61.7) | | 49.4  (39.8-61.5) | | 0.34 |
| Stratify age |  |  |  |  | 0.99 |
| ≤49 | 7087 | 51.5 | 1773 | 51.5 |  |
| 50-65 | 3889 | 28.3 | 973 | 28.3 |  |
| >65 | 2784 | 20.2 | 696 | 20.2 |  |
| Gender |  |  |  |  | 0.99 |
| Women | 7357 | 53.5 | 1840 | 53.5 |  |
| Men | 6403 | 46.5 | 1602 | 46.5 |  |
| Comorbidity |  |  |  |  |  |
| Hypertension | 5546 | 40.3 | 1388 | 40.3 | 0.98 |
| Diabetes | 1617 | 11.8 | 406 | 11.8 | 0.94 |
| Hyperlipidemia | 1064 | 7.73 | 268 | 7.79 | 0.92 |
| Gout | 457 | 3.32 | 116 | 3.37 | 0.89 |
| Urinary tract infection | 1084 | 7.88 | 272 | 7.90 | 0.96 |
| Obesity | 59 | 0.43 | 16 | 0.46 | 0.77 |
| Chronic kidney disease | 177 | 1.29 | 45 | 1.31 | 0.92 |
| Fracture | 1183 | 8.60 | 266 | 7.73 | 0.10 |
| Hematuria | 99 | 0.72 | 17 | 0.49 | 0.15 |

Chi-square test; *t*-test

Abbreviations: IQR, interquartile range; PA, primary aldosteronism.

Supplementary Table 2. Comparison of bladder stone risk in incidence densities, hazard ratio, and sub-hazard ratio in a competing risk (death) model between patients with and without PA stratified by demographic characteristics and comorbidity

|  | Primary Aldosteronism | | | | | |  |  |  |  |  |  |
| --- | --- | --- | --- | --- | --- | --- | --- | --- | --- | --- | --- | --- |
|  | No  (n=13760) | | | Yes  (n=3442) | | |  |  |  |  |  |  |
|  | Event | PY | Rate^#^ | Event | PY | Rate^#^ | Crude HR^a^  (95 % CI) | Adjusted HR^b^ (95 % CI) | p-value | Crude SHR^†^  (95 % CI) | Adjusted SHR^＆^ (95 % CI) | p-value |
|  |  |  |  |  |  |  |  |  |  |  |  |  |
| All | 232 | 69181 | 3.35 | 90 | 16786 | 5.36 | 1.60(1.25, 2.04) | 1.63(1.28, 2.08) | <0.001 | 1.59(1.25, 2.02) | 1.57(1.24, 2.00) | <0.001 |
| Age |  |  |  |  |  |  |  |  |  |  |  |  |
| 20-49 | 98 | 39311 | 2.49 | 45 | 9872 | 4.56 | 1.83(1.29, 2.61) | 1.86(1.31, 2.65) | <0.001 | 1.57(1.11, 2.21) | 1.57(1.11, 2.21) | 0.01 |
| 50-64 | 86 | 18602 | 4.62 | 25 | 4416 | 5.66 | 1.22(0.78, 1.90) | 1.23(0.78, 1.91) | 0.37 | 1.26(0.81, 1.97) | 1.25(0.80, 1.96) | 0.32 |
| 65+ | 48 | 11267 | 4.26 | 20 | 2498 | 8.00 | 1.86(1.10, 3.13) | 1.90(1.13, 3.20) | 0.02 | 1.92(1.14, 3.22) | 1.94(1.15, 3.26) | 0.01 |
| Gender |  |  |  |  |  |  |  |  |  |  |  |  |
| Women | 101 | 37782 | 2.67 | 37 | 9234 | 4.01 | 1.50(1.03, 2.19) | 1.55(1.06, 2.26) | 0.02 | 1.48(1.02, 2.14) | 1.45(1.00, 2.11) | 0.04 |
| Men | 131 | 31399 | 4.17 | 53 | 7552 | 7/02 | 1.68(1.22, 2.31) | 1.70(1.24, 2.34) | 0.001 | 1.69(1.23, 2.33) | 1.70(1.23, 2.33) | 0.001 |
| Comorbidity^§^ |  |  |  |  |  |  |  |  |  |  |  |  |
| No | 95 | 39248 | 2.42 | 47 | 9517 | 4.94 | 2.04(1.44, 2.90) | 2.08(1.47, 2.95) | <0.001 | 1.82(1.30, 2.55) | 1.82(1.30, 2.55) | <0.001 |
| Yes | 137 | 29933 | 4.58 | 43 | 7269 | 5.92 | 1.29(0.92, 1.82) | 1.31(0.93, 1.84) | 0.12 | 1.35(0.96, 1.90) | 1.32(0.94, 1.86) | 0.11 |

Rate^#^, incidence rate, per 1000 person-years.

Crude HR^a^, relative hazard ratio.

Adjusted HR^b^, adjusted hazard ratio, was calculated by Cox model and adjusted for age, sex, and comorbidities of hypertension, diabetes, hyperlipidemia, gout, urinary tract infection, obesity, chronic kidney disease, fracture, and hematuria.

Crude SHR^†^, relative sub-hazard ratio.

Adjusted SHR^＆^, adjusted sub-hazard ratio, was calculated by competing risk (death) model and adjusted for age, sex, and comorbidities of hypertension, diabetes, hyperlipidemia, gout, urinary tract infection, obesity, chronic kidney disease, fracture, and hematuria.

Comorbidity^§^: Patients with any one of the comorbidities (including hypertension, diabetes, hyperlipidemia, gout, urinary tract infection, obesity, chronic kidney disease, fracture, and hematuria) were classified as the group of comorbidity.

Abbreviations: CI, confidence interval; HR, hazard ratio; PY, SHR, sub-hazard ratio.
